# Autologous Conditioned Serum in Knee Osteoarthritis: A Systematic Review of Current Clinical Evidence

**DOI:** 10.7759/cureus.68963

**Published:** 2024-09-08

**Authors:** Naveen Jeyaraman, Madhan Jeyaraman, Swaminathan Ramasubramanian, Sankalp Yadav, Sangeetha Balaji, Bishnu P Patro, Ashim Gupta

**Affiliations:** 1 Orthopaedics, ACS Medical College and Hospital, Dr MGR Educational and Research Institute, Chennai, IND; 2 Orthopaedics, South Texas Orthopaedic Research Institute (STORI), Laredo, USA; 3 Clinical Research, Viriginia Tech India, Dr MGR Educational and Research Institute, Chennai, IND; 4 Orthopaedics, Government Medical College, Omandurar Government Estate, Chennai, IND; 5 Medicine, Shri Madan Lal Khurana Chest Clinic, New Delhi, IND; 6 Orthopaedics, All India Institute of Medical Sciences, Bhubaneswar, IND; 7 Regenerative Medicine, Future Biologics, Lawrenceville, USA; 8 Regenerative Medicine, BioIntegrate, Lawrenceville, USA

**Keywords:** autologous conditioned serum, knee, osteoarthritis, platelet-rich plasma (prp), regenerative medicine

## Abstract

Knee osteoarthritis (OA) significantly impacts global health, causing pain, disability, and socioeconomic burden. Traditional treatments often provide only temporary relief and can have adverse effects. Autologous conditioned serum (ACS) therapy, which enriches a patient's own blood with growth factors and anti-inflammatory cytokines, has emerged as a promising approach to manage knee OA, potentially offering pain reduction, improved function, and tissue regeneration. Following Preferred Reporting Items for Systematic Reviews and Meta-Analyses (PRISMA) guidelines, we searched databases such as PubMed, Web of Science, and Cochrane using terms like "Autologous Conditioned Serum" and "knee osteoarthritis." Clinical studies were selected based on their focus on ACS's efficacy in knee OA, assessing outcomes like pain relief, functional improvement, and adverse events. Eighteen studies met the inclusion criteria, including randomized controlled trials, observational studies, and comparative analyses. The review included a wide range of study designs and outcomes, highlighting ACS's efficacy in reducing pain and enhancing knee function as evidenced by various patient-reported outcome measures Visual Analog Scale (VAS), Western Ontario and McMaster Universities Osteoarthritis Index (WOMAC), Knee Injury and Osteoarthritis Outcome Score (KOOS), Knee Society Clinical Rating Score (KSCRS) with a follow-up of up to 11 years (range: 2-11 years). Comparative studies showed ACS to be as effective or superior to conventional treatments such as platelet-rich plasma, steroids, and hyaluronic acid, especially in cases of moderate synovitis. Minimal adverse effects such as peri-injection pain, rigidity, synovitis, transient sensation of redness/heat, and numbness in the knee/leg/toes were reported, underscoring ACS's safety. Some studies suggested ACS might also have disease-modifying effects, contributing to tissue repair and integrity. ACS therapy offers a promising alternative for knee OA management, demonstrating potential benefits in symptom alleviation, functional improvement, and safety. Indications of disease-modifying properties further highlight its therapeutic value. However, the need for standardized formulations and treatment protocols, long-term studies, and mechanistic understanding remain. Future research should focus on addressing these gaps to fully elucidate ACS's role in the treatment landscape of knee OA.

## Introduction and background

Osteoarthritis (OA) of the knee represents a predominant cause of musculoskeletal disability globally, impacting millions with pain and functional limitation [1]. Characterized by the progressive erosion of articular cartilage, alongside alterations in subchondral bone and synovial inflammation, knee OA not only diminishes life quality but also imposes a significant socioeconomic burden [2,3]. Traditional therapeutic approaches, primarily aimed at alleviating symptoms, include non-steroidal anti-inflammatory drugs (NSAIDs), analgesics, and intra-articular corticosteroid and hyaluronic acid injections [4-8]. However, these strategies often yield only temporary relief and carry the potential for adverse effects, underscoring the imperative for innovative interventions that target disease modification or promote regenerative healing [9].

Emerging as a promising modality, autologous conditioned serum (ACS) therapy has garnered interest for its potential in knee OA management. This technique involves the procurement and processing of the patient's own blood to enrich it in growth factors and anti-inflammatory cytokines, which are subsequently reintroduced into the affected knee. The theoretical foundation of ACS therapy is its capacity to modulate the intra-articular environment, mitigating inflammation and fostering repair mechanisms. Preliminary evidence from clinical and preclinical studies suggests that ACS may enhance knee function, diminish pain, and possibly encourage tissue regeneration, offering a novel avenue for knee OA treatment [9,10]. In the context of emerging therapies for knee OA, it is crucial to distinguish ACS from other autologous blood products, particularly platelet-rich plasma (PRP). While both are derived from the patient's own blood, they differ significantly in preparation and composition. ACS is produced by incubating whole blood with medical-grade glass beads, inducing leukocytes to generate anti-inflammatory cytokines and growth factors, particularly interleukin-1 receptor antagonists (IL-1Ra) [11]. In contrast, PRP is created by concentrating platelets through centrifugation, resulting in a high concentration of platelet-derived growth factors. This fundamental difference leads to distinct proposed mechanisms of action: ACS primarily aims to modulate the inflammatory environment within the joint, potentially offering disease-modifying effects [12], while PRP focuses more on stimulating tissue repair and regeneration. Understanding these distinctions is essential for interpreting the comparative efficacy of ACS in knee OA management and for guiding future research directions. Despite the encouraging prospects of ACS for knee OA, significant knowledge gaps persist. The underlying mechanisms driving its therapeutic benefits remain inadequately elucidated, and patient responses to treatment exhibit variability. Moreover, comprehensive evaluations of the long-term efficacy and safety of ACS injections are lacking.

The primary objective of this systematic review is to assess the efficacy of ACS in treating knee OA. By synthesizing evidence from clinical studies, this review aims to clarify ACS's effects on symptom alleviation, knee function enhancement, and potential tissue repair. Furthermore, it seeks to identify determinants of treatment success and to appraise the safety profile of ACS therapy. Through this comprehensive evaluation, we endeavor to offer evidence-based insights that could influence clinical practice and guide future investigatory paths, ultimately advancing the management of knee OA and improving patient outcomes and overall quality of life.

## Review

Materials and methods

Search Strategy and Study Selection

**Figure 1:**
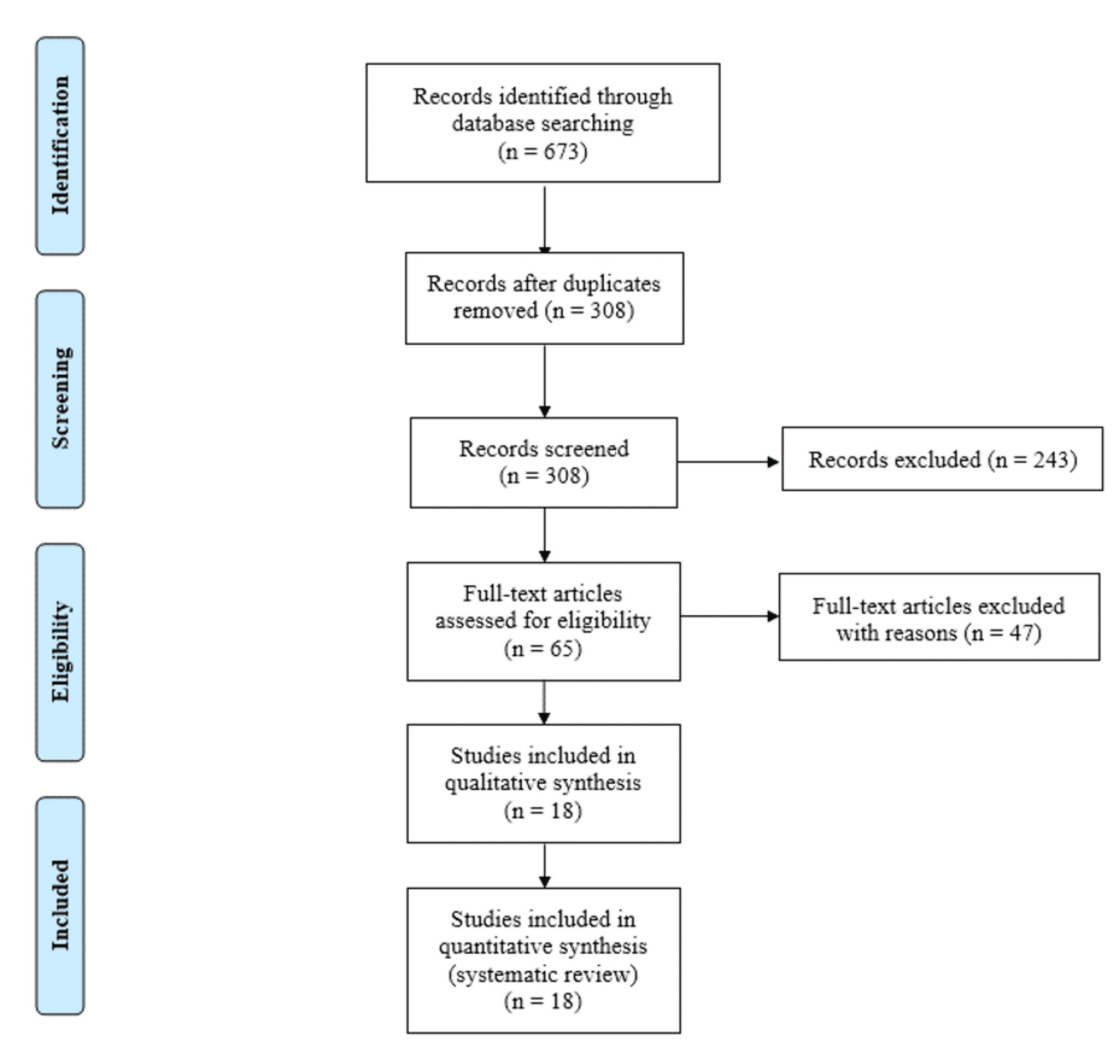
PRISMA flowchart. PRISMA: Preferred Reporting Items for Systematic Reviews and Meta-Analyses

The methodology of the systematic review adhered to the Preferred Reporting Items for Systematic Reviews and Meta-Analyses (PRISMA) guidelines. An exhaustive search strategy was implemented across multiple electronic databases, including PubMed, PubMed Central, Web of Science, and Cochrane. The search terms used were "Autologous Conditioned Serum" OR “ACS” and "osteoarthritis, knee". These terms were selected to encompass the various nomenclatures associated with ACS and to specifically focus on the application of ACS in knee OA. The initial search identified a total of 673 records. To ensure the relevancy and focus of the systematic review, duplicate records were removed, yielding 308 unique studies for consideration (Figure 1).

Screening and Eligibility Assessment

The screening process involved a meticulous review of titles and abstracts, resulting in the exclusion of 243 records that did not meet the inclusion criteria or were irrelevant to the research questions. This process was conducted by two independent reviewers, with discrepancies resolved through discussion or consultation with a third reviewer. Subsequently, 65 full-text articles were assessed for eligibility, leading to the exclusion of 47 articles with reasons such as lack of relevance to the research question, insufficient data on ACS treatment outcomes, or poor study quality. Studies not explicitly examining the effects of ACS on knee OA were also excluded. PRISMA flow diagram is shown in Figure 1.

Inclusion Criteria

The selection criteria for studies encompassed several key parameters. Firstly, inclusion relied on the utilization of ACS, as an intervention for knee OA. Additionally, studies needed to incorporate functional outcomes, notably pain levels and joint mobility, as primary measures of effectiveness.

Exclusion Criteria

Investigations incorporating orthobiologic modalities distinct from ACS were systematically excluded. Furthermore, research employing ACS for applications divergent from KOA was also methodically excluded.

Data Extraction and Quality Assessment

Eighteen studies met the inclusion criteria and were subjected to qualitative synthesis. Data extracted from these studies included author names, year of publication, study design, number of participants, Kellgren-Lawrence (KL) grade, dosage of ACS, functional outcomes with follow-up periods, complications, and the significance of the findings.

Results

The systematic review incorporated a diverse assortment of studies evaluating the effectiveness of ACS in managing knee OA, featuring various designs, participant volumes, and outcomes. Across the 18 studies reviewed, the participant count varied from 15 to 126, reflecting a wide scope of research dimensions and contexts.

Observational and prospective investigations, as well as randomized control trials, underscored the efficacy of ACS in enhancing functional outcomes such as the Visual Analog Scale (VAS), Western Ontario and McMaster Universities Osteoarthritis Index (WOMAC), Knee Society Clinical Rating Scale (KSCRS), and Knee injury and Osteoarthritis Outcome Score (KOOS) over different follow-up intervals. Notably, García-Escudero et al. and Barreto et al. reported substantial improvements in VAS and WOMAC scores, persisting for up to two years, highlighting the long-term relief ACS may offer knee OA patients [11,12].

Comparative analyses provided insights into ACS's relative performance against treatments like PRP, steroids, and hyaluronic acid. Studies by Shirokova et al. [13], Coşkun et al. [14], and Cheng et al. [15] showcased ACS's efficacy, either surpassing or on par with these modalities, especially in cases of moderate synovitis and superior pain management and functional results compared to PRP. A notable observation across the studies was the minimal reporting of adverse effects, underscoring the safety of ACS treatments. While minor complications such as peri-injection discomfort and transient symptoms were noted in some instances, these did not significantly impact the overall positive safety and tolerability profile of ACS interventions. Moreover, research by Vitali et al. [16], Baltzer et al. [17], and Hashemi et al. [18] posited ACS as a potential disease-modifying intervention for knee OA, suggesting its utility beyond mere symptom alleviation to contributing to the structural integrity of knee joints. The results are tabulated in Table 1.

**Table 1:** Summary of the included studies in the systematic review ACS: Autologous Conditioned Serum; KSCRS/KSS: Knee Society Clinical Rating Scale; VAS: Visual Analog Pain Scale-; XSMFA-D: Extra Short Musculoskeletal Functional Assessment Survey; PGIC: Patient Global Impression of Change Survey; IA: Intra-articular; KOA: Knee Osteoarthritis; WOMAC: Western Ontario and McMaster Universities Arthritis Index; KOOS: Knee Injury and Osteoarthritis Outcome Score; US: Ultrasound; BMAC: Bone Marrow Aspirate Concentrate; PRP: Platelet-Rich Plasma; ROM: Range of Motion; NRS: Numeric Rate Scale; KL: Kellgren-Lawrence

Author, year	Type of study	Number of participants	KL grade	Dosage	Functional outcome and follow-up	Complications	Significance
Baltzer et al., 2009 [17]	Double-blinded randomized control trial	ACS 126 patients; hyaluronic acid 120 patients; and saline 99 patients	II – III	IA ACS (2 mL) injection every week for six weeks; IA hyaluronic acid injection every week for six weeks; and IA saline (2 mL) injection every week for six weeks	WOMAC, global patient assessment, VAS, and Short-Form 8 for 24 months	No major adverse effects reported	ACS appears to be disease-modifying, chondroprotective, and chondro-regenerative in knee OA.
García-Escudero et al., 2015 [11]	Observational study	118 patients (118 knees)	I – IV	IA ACS (2 mL) injections once weekly over four weeks and subsequent physiotherapy applied four weeks after ACS injection	VAS and WOMAC scores for two years	1 patient resulted in total knee replacement	Combination of ACS and physiotherapy demonstrated an improvement in VAS and WOMAC scores for two years in patients with knee OA
Khurana et al., 2021 [19]	Comparative study	PRP 27 patients; ACS 21 patients; steroid 28 patients; and hyaluronic acid 20 patients	I – III	IA 5 mL PRP injection; IA 6 mL ACS injection; IA 40 mg depot-methylprednisolone mixed with 5 mL of 2% lidocaine hydrochloride with 1:80,000 epinephrine; and IA 6 mL hyaluronic acid	VAS and WOMAC scores for six months	Not specified	Clinical outcomes following IA ACS and PRP are better than controls; Failed to demonstrate the clinical significance between the two orthobiologics.
Tassara et al., 2018 [20]	Retrospective study	25 patients	Not specified	IA ACS (2 mL) injections every week for four weeks	VAS and range of motion for six months	No adverse effects reported	ACS demonstrated rapid pain relief and improved ROM drastically in patients with knee OA.
Vitali et al., 2022 [21]	Comparative analysis	BMAC 12 patients and ACS 12 patients	I – III	IA BMAC (7 – 10 mL) injections and IA ACS (3 mL) injections every week for four weeks	VAS, WOMAC, and ROM for six months	No adverse effects reported	BMAC demonstrated superior clinical efficacy than ACS injection in knee OA.
Leone et al., 2021 [22]	Observational study	30 patients	I – III	IA ACS (2 mL) injections every week for four weeks	VAS for one year	Pain and synovitis reported in few patients	ACS demonstrates a better clinical efficacy in knee OA resistant to medical management and PRP injection
Barreto et al., 2017 [12]	Prospective observational study	100 patients	Not defined	US-guided IA injections of ACS on day 0, 7, 14, 90, 180, and 270	VAS, XSMFA-D, PGIC for one year	8 patients resulted with no improvement in pain and functional scores following ACS injection	Arthrokinex^TM^ (ACS) is a safe, point-of-care alternative to provide significant clinical benefits in patients with symptomatic knee OA.
Abd-EL Motaal et al., 2014 [23]	Prospective study	30 knees	I – III	IA ACS (1 mL) for once a week for three weeks	WOMAC for six months	Pain and swelling were reported	ACS (IL-1RA) is a viable treatment option for knee OA.
Pishgahi et al., 2020 [24]	Randomized clinical trial	Prolotherapy 30 patients; PRP 30 patients; and ACS 32 patients	II – IV	US-guided IA 2 mL of 50% dextrose, 1 mL of 2% lidocaine, and 2 mL of distilled water every week for three weeks; US-guided IA PRP (2 mL) injection twice a week for two weeks; and US-guided IA ACS (2 mL) injection twice a week for two weeks	VAS and WOMAC scores for six months	No adverse effects reported	ACS showed improved pain scores and functional outcomes in knee OA when compared with PRP and prolotherapy
Damjanov and Zekovic, 2023 [25]	Double-blinded randomized control trial	ACS 16 patients and Placebo 17 patients	III – IV	1 ml of 40 mg triamcinolone followed by IA ACS (5 mL) injection IA saline placebo (5 mL) injection	NRS and KOOS scores for two years	Transient sensation of redness/heat, increase in blood pressure, transient increase in pain, and numbness in knee/leg/toes.	ACS provides long-term pain relief and improved functional outcomes when compared with other modalities in knee OA.
Zarringam et al., 2018 [26]	Prospective cohort study	Orthokin 72 patients and placebo 54 patients	Not specified	Not specified	11 years	Not specified	Orthokin for knee OA does not delay surgical management for advanced stage of knee OA
Kilinç and Öç, 2019 [27]	Retrospective study	33 patients	II – III	IA ACS (2 mL) twice a week for three weeks	VAS, KOOS, and KSS scores for one year	No adverse effects reported	ACS is effective in low- to medium-grade knee OA.
Shirokova et al., 2020 [13]	Prospective, controlled, open-label clinical study in	123 patients into two groups	II – III	Six IA injections of ACS (2.5 mL) twice per week and six IA injections of PRP (5 mL) twice per week	VAS and WOMAC scores for three months	No adverse effects reported	ACS exerted a differential therapeutic effect in knee OA with superiority over PRP in case of moderate synovitis.
Vitali et al.,2020 [16]	Observational study	15 patients	I – III	US-guided IA ACS injections every week for four weeks	VAS, WOMAC, and KSS scores for six months	Peri-injection pain and rigidity (n=1) reported	ACS acts as disease-modifying osteo-arthritis drugs for knee OA.
Hashemi et al., 2020 [18]	Double-blinded randomized control trial	ACS 30 patients and ozone 30 patients	I – IV	IA ACS (2 mL) injection on 1^st^, 7^th^, 14^th^, and 21^st^ days and IA 10 mL ozone (30 μg/mL + 5 mL of lidocaine 1%) injection on 1^st^, 7^th^, 14^th^, and 21^st^ days	VAS, WOMAC, and KOOS for six months	No major adverse effects reported	Clinically significant improvement was observed with ACS in knee OA
Coşkun et al., 2022 [14]	Retrospective study	ACS 40 patients and PRP 42 patients	II – III	IA ACS (3 mL) injections every week for three weeks and IA PRP (3 mL) injections every week for three weeks	VAS and KOOS scores for five years	Adverse effects were noted in 5% of patients in ACS group and 38.1% of patients in PRP group	Effectiveness of ACS and PRP declines after two years of follow-up; ACS demonstrated superior pain relief and functional outcome when compared with PRP.
Cheng et al., 2023 [15]	Comparative analysis	PRP 30 patients and ACS 30 patients	III	Five doses of IA PRP injection every two-weekly interval and 5 doses of IA ACS injection every two-weekly interval	VAS	No adverse effects reported	ACS provided better improvement in pain relief compared to PRP in knee OA.

Discussion

ACS represents an innovative treatment avenue for knee OA, a leading cause of disability and pain worldwide. With traditional treatment modalities often limited to symptomatic relief and associated with various side effects, the exploration of ACS offers a potentially transformative approach. This serum, enriched with anti-inflammatory cytokines and growth factors derived from the patient's own blood, has shown promise in not only alleviating symptoms but also in modifying the disease process. This detailed discussion draws from a systematic review of diverse studies, shedding light on the efficacy, safety, and potential disease-modifying properties of ACS in knee OA treatment.

ACS's comparative effectiveness against established treatments like PRP, steroids, and hyaluronic acid has been a focal point of several studies. Shirokova et al. [13] and Cheng et al. [15] highlighted ACS's superiority or comparability, with particular effectiveness noted against moderate synovitis and superior pain management outcomes compared to PRP, as documented by Coskun et al. [14]. This is pivotal as it suggests ACS may offer a more robust solution in specific knee OA subgroups, potentially guiding more personalized treatment strategies. Furthermore, the improvement in functional outcomes, as evidenced by VAS, WOMAC, KOOS, and KSCRS scores across studies, underscores ACS's role in enhancing the quality of life for knee OA patients. The significant long-term improvements reported by García-Escudero et al. [11] and Barreto et al. [12] underscore the potential of ACS to provide sustained benefits, challenging the transient relief commonly associated with traditional OA treatments. Khurana et al. [19] delve into the comparative effectiveness of ACS against other orthobiologics and conventional treatments like steroids and hyaluronic acid. Their research underscores the relative superiority of ACS in clinical outcomes, albeit without establishing a marked distinction between ACS and PRP, which prompts a call for further nuanced exploration into the specific contexts where ACS may offer the most benefit. Tassara et al. [20] elucidate ACS's rapid analgesic effects and enhancement of joint mobility, further establishing ACS as a potent intervention for pain relief and functional improvement in knee OA patients. This retrospective study highlights the significance of ACS in providing quick symptomatic relief and enhancing the quality of life.

The minimal adverse effects reported across the studies underline ACS's safety, making it a viable option for patients where other treatments might pose significant risks. The absence of major adverse effects, as noted in studies by Shirokova et al. [13] and Vitali et al. [16], is encouraging, emphasizing ACS's tolerability and potential for wider clinical adoption. The review intriguingly suggests ACS's capacity to act beyond symptom management, venturing into disease modification. The findings from Vitali et al. [16] and Baltzer et al. [17] highlight its chondroprotective and regenerative capabilities. This positions ACS as not just a treatment but a potential agent in altering the disease trajectory of knee OA, an aspect that markedly differentiates it from existing therapies. Vitali et al. [21] and Leone et al. [22] both provide insights into the comparative and specific efficacies of ACS in treating knee OA.

Vitali et al.'s comparative analysis with bone marrow aspirate concentrate (BMAC) positions ACS in the broader context of regenerative treatments, suggesting areas where ACS might be optimized or combined with other modalities for enhanced outcomes. Leone et al. spotlight ACS as a particularly effective treatment for cases resistant to conventional medical management and PRP injections, emphasizing its role in tackling more challenging knee OA instances [22]. Abd-EL Motaal et al. further corroborate the effectiveness of ACS in managing knee OA, showcasing the viability of ACS as a treatment option, especially focusing on its anti-inflammatory properties [23]. Hashemi et al. [18] and Pishgahi et al. [24] both underscore the clinical significance and improvement observed with ACS treatment in comparison to other modalities like ozone and prolotherapy. These studies augment the evidence base supporting ACS's efficacy, particularly highlighting its role in improving pain scores and functional outcomes, hence contributing valuable insights for clinicians in formulating personalized treatment plans.

The quest for understanding the mechanisms underlying ACS's effects is paramount. As Damjanov et al. pointed out, while ACS provides long-term relief, deciphering its biological underpinnings will be crucial for optimizing its therapeutic potential [25]. This necessitates further research, particularly randomized controlled trials with long-term follow-up, to establish standardized treatment protocols, ascertain the optimal dosage and administration frequency, and elucidate the regenerative capabilities of ACS in knee cartilage. Zarringam et al. contribute a critical perspective by examining the long-term implications of ACS treatment on surgical intervention timelines [26]. Their findings suggest that while ACS offers therapeutic benefits, it may not significantly delay the need for surgical management in advanced knee OA stages, thus adding an essential dimension to understanding the scope and limitations of ACS therapy. Kilinç and Öç further corroborate the effectiveness of ACS in managing knee OA, by noting its efficacy in low- to medium-grade knee OA [27]. The variability in treatment responses, as illustrated by Rutgers et al. [28], and the absence of a standardized protocol present challenges that need addressing. This variability underscores the necessity for individualized treatment approaches and further elucidates the complexity of knee OA as a multifactorial disease.

ACS emerges as a promising modality in the knee OA treatment landscape, offering a blend of symptom relief, safety, and potential disease modification. The journey from experimental therapy to a staple in clinical practice will require rigorous research efforts, aiming to unravel the complexities of its mechanism, optimize treatment protocols, and fully ascertain its long-term efficacy and safety. As such, ACS stands at the precipice of potentially redefining knee OA management, warranting further exploration and validation in the clinical realm.

Despite the promising results observed in the efficacy and safety of ACS in the management of knee OA, this systematic review has encountered several limitations that warrant careful consideration. The included studies exhibit a broad range of designs, from observational studies and prospective cohort studies to randomized controlled trials, which complicates the direct comparison of outcomes and the pooling of data for meta-analyses, potentially affecting the generalizability of the findings. The lack of standardization in the preparation, dosage, and administration schedule of ACS across studies introduces a significant variable that could influence the efficacy and safety outcomes, making it challenging to ascertain the optimal therapeutic protocol for ACS in knee OA treatment. Many studies included in this review have relatively short follow-up periods, limiting the ability to assess the long-term efficacy and safety of ACS treatment, and the sustainability of symptomatic relief and functional improvement over time remains uncertain. The biological mechanisms underlying the therapeutic effects of ACS on knee OA are not fully elucidated within the reviewed literature, and a deeper understanding of the molecular and cellular interactions induced by ACS is crucial for optimizing its therapeutic potential and for identifying patient subgroups that may benefit the most. There is notable variability in the outcomes reported across studies, including different scales for pain, function, and quality of life, as well as diverse measures of structural change, which complicates the synthesis of evidence and the formulation of definitive conclusions regarding the efficacy of ACS. The possibility of publication bias, where studies with positive findings are more likely to be published, cannot be overlooked, and the comprehensive assessment of safety is constrained by the underreporting of complications and side effects, particularly in studies with smaller sample sizes and shorter follow-up periods.

Given the limitations outlined above, several avenues for future research emerge, highlighting the need for a concerted effort to enhance the evidence base surrounding ACS therapy for knee OA. Developing and adhering to standardized protocols for the preparation, dosage, and administration of ACS is paramount, as this would facilitate the direct comparison of study outcomes and aid in establishing optimal treatment regimens. Conducting long-duration randomized controlled trials with extended follow-up periods is essential to comprehensively assess the long-term efficacy, safety, and potential disease-modifying effects of ACS in knee OA patients. Investing in basic science and translational research to unravel the molecular mechanisms of action of ACS can provide insights into its therapeutic potential, guide treatment personalization, and identify biomarkers predictive of response. Head-to-head comparisons of ACS with other biologic therapies such as PRP and stem cell therapies, within well-designed clinical trials, would clarify its relative effectiveness and position within the treatment algorithm for knee OA. Identifying patient characteristics and disease phenotypes that predict a favorable response to ACS treatment could enable a more targeted and personalized approach to therapy, enhancing outcomes and cost-effectiveness. Systematic collection and analysis of safety data, including rare and long-term adverse effects, are crucial for a balanced assessment of the risk-benefit profile of ACS therapy. Finally, conducting cost-effectiveness analyses alongside clinical trials would provide valuable information on the economic viability of ACS as a treatment option for knee OA, considering the growing emphasis on value-based care in healthcare decision-making. Rigorous, long-duration randomized controlled trials are essential to solidify ACS's role in knee OA therapy and to refine treatment methodologies.

## Conclusions

This systematic review identifies ACS as a promising treatment for knee OA, demonstrating effectiveness in reducing symptoms, improving function, and potentially modifying disease progression with minimal side effects. While the evidence suggests ACS could significantly enhance patient quality of life, this review emphasizes the need for standardized protocols, long-term randomized trials, and deeper exploration of its biological mechanisms. Addressing these gaps is crucial to confirm the long-term efficacy and safety of ACS and to determine its optimal role in treating knee OA.
